# EMPATHIC RESPONSE AND MARITAL QUALITY FOR SAME- AND DIFFERENT-SEX MIDLIFE COUPLES: MEDIATING ROLE OF DYADIC COPING

**DOI:** 10.1093/geroni/igac059.3058

**Published:** 2022-12-20

**Authors:** Susanna Joo, Hye Won Chai, Jihye Lee, Hyoun K Kim, Debra Umberson

**Affiliations:** Yonsei University, Seoul, Seoul-t’ukpyolsi, Republic of Korea; The University of Texas at Austin, Austin, Texas, United States; Yonsei University, Seoul, Seoul-t’ukpyolsi, Republic of Korea; Yonsei University, Seoul, Seoul-t’ukpyolsi, Republic of Korea; The University of Texas at Austin, Austin, Texas, United States

## Abstract

Dyadic coping is a daily interpersonal process that married couples use to manage stress and maintain their marriage. However, little is known about its mediating role in the association between empathic response and marital quality among same-sex and different-sex couples. This study aimed to examine the extent to which dyadic coping mediates the association between empathic response and marital quality, focusing on middle-aged men and women in same-sex and different-sex marriages. We used dyadic data from the Health and Relationships Project (HARP), including 124 gay, 171 lesbian, and 124 straight couples. Results from the actor-partner interdependence mediation model (APIMeM) showed that dyadic coping within couples mediated the association between empathic response and marital quality for all couple types (i.e., gay, lesbian, and straight couples). More empathic response was associated with better dyadic coping, which led to higher marital quality. While such mediated paths did not differ significantly between gay and lesbian couples, direct associations between empathic responses and marital quality were only significant among lesbian couples. Additionally, there were gendered patterns within straight couples; while female spouses’ empathic response was associated with their and their male spouses’ marital quality through the couple’s dyadic coping, such a mediated path was not significant for male spouses’ empathic response. These findings suggest dyadic coping as an effective strategy for enhancing marital quality among same-sex and different-sex married couples, but the mediating role of dyadic coping is gendered in different-sex marriages.

